# The Effect of Antivascular Endothelial Growth Factor Therapy on the Development of Neovascular Glaucoma after Central Retinal Vein Occlusion: A Retrospective Analysis

**DOI:** 10.1155/2014/317694

**Published:** 2014-04-01

**Authors:** Christina L. Ryu, Adrian Elfersy, Uday Desai, Thomas Hessburg, Paul Edwards, Hua Gao

**Affiliations:** ^1^Department of Ophthalmology, Henry Ford Hospital, 2799 W. Grand Boulevard, Detroit, MI 48202, USA; ^2^Department of Ophthalmology, Henry Ford Medical Center, 6530 Farmington Road, West Bloomfield, MI 48322, USA

## Abstract

*Purpose*. Ischemic central retinal vein occlusion (CRVO) eyes are at high risk of developing neovascular glaucoma (NVG). Our purpose is to investigate the effect of anti-VEGF therapy for macular edema after CRVO on the development of neovascular glaucoma (NVG) in ischemic CRVO eyes. *Methods*. This is a retrospective case series of 44 eyes from 44 patients with CRVO treated with anti-VEGF therapy for macular edema. The primary outcome was the development of NVG. *Results*. Of the 44 eyes, 14 eyes had ischemic CRVO, and 30 eyes had nonischemic CRVO. Nonischemic eyes received a mean of 8.4 anti-VEGF doses, over mean follow-up of 24 months. One nonischemic eye (3.3%) developed NVD but not NVG. The 14 ischemic eyes received a mean of 5.6 anti-VEGF doses, with mean follow-up of 23 months. Of these 14 ischemic eyes, two eyes (14%) developed iris neovascularization and 3 eyes (21%) developed posterior neovascularization. Three of these 5 eyes with neovascularization progressed to NVG, at 19.7 months after symptom onset, on average. *Conclusion*. Anti-VEGF therapy for macular edema may delay, but does not prevent, the development of ocular NV in ischemic CRVO. Significant risk of NVG still exists for ischemic CRVO eyes.

## 1. Introduction

Central retinal vein occlusion (CRVO) is a common sight-threatening retinal vascular disorder, with a cumulative 10-year incidence of 1.6% [1]. CRVO is classified into ischemic or nonischemic type, with approximately 20% of CRVO cases presenting as ischemic type [2, 3]. Ischemic CRVO is associated with significantly poorer visual outcomes and a high risk of ocular neovascularization (NV) [2, 4]. If left untreated, ocular NV from ischemic CRVO often leads to neovascular glaucoma (NVG), with the incidence in an untreated ischemic eye ranging from 23% to 60% [5]. The phrase “90-day glaucoma” has been coined to describe the development of NVG secondary to ischemic CRVO. In the Central Vein Occlusion Study (CVOS), panretinal photocoagulation (PRP) was recommended to treat ocular NV after it develops, but prophylactic PRP did not reduce the rate of neovascularization [2].

Ocular NV results from retinal ischemia, with vascular endothelial growth factor (VEGF) playing a key role in the development of pathological angiogenesis [6, 7]. It has been reported that bevacizumab injection is very effective in the treatment of iris neovascularization and as an adjunct in the treatment of NVG in CRVO eyes [8–10]. Recent studies suggested a protective effect of anti-VEGF against neovascularization after retinal vein occlusion [11, 12]. Over the past few years, anti-VEGF agents ranibizumab (Lucentis, Genentech, Inc., South San Francisco, CA), bevacizumab (Avastin, Genentech, Inc., South San Francisco, CA), and more recently aflibercept (Eylea, Regeneron, Tarrytown, NY) have become widely used for the treatment of macular edema from CRVO, resulting in significant visual improvement [13–15]. Herein we report a retrospective series of ischemic and nonischemic CRVO eyes with macular edema treated with anti-VEGF agents to investigate whether anti-VEGF therapy may reduce the development of NVG and ocular NV.

## 2. Methods

After receiving approval by the Institutional Research Board at our institution, we reviewed eyes treated with an anti-VEGF agent (ranibizumab or bevacizumab) for macular edema secondary to CRVO. Patients were evaluated and treated by one of the four retina specialists at our large urban institution from August 2007 to March 2012. Cases were screened and identified through review of CPT codes for anti-VEGF agent and ICD-9 codes for “central retinal vein occlusion,” “venous tributary occlusion,” and “retinal vascular occlusion not otherwise specified.” A control group was not utilized in this retrospective study as the natural history of CRVO has been well elucidated by prior studies and the CVOS [2, 4, 5].

Patients with macular edema due to CRVO with symptom onset no greater than 90 days prior to presentation were included, of both ischemic and nonischemic CRVO types. Patients with medication-controlled open angle glaucoma and dry age-related macular degeneration were included. Exclusion criteria were the presence of NVG or any ocular NV on initial presentation, previous PRP, or a history of proliferative diabetic retinopathy. Patients with follow-up duration of less than 8 months were excluded. The data was compiled from 44 eyes of 44 patients with CRVO that fulfilled the inclusion criteria.

Patients were diagnosed as ischemic versus nonischemic CRVO by the treating retina specialist or, if this distinction was not documented in the patient's record, by the investigators based on well-established criteria: the presence of relative afferent papillary defect (RAPD), visual acuity (20/200 or worse), and the extent of nonperfusion on fluorescein angiography, as previously reported [3]. Macular edema was diagnosed and quantified with Zeiss Stratus optical coherence tomography (OCT) or Zeiss Cirrus HD OCT (Carl Zeiss Meditec, Jena, Germany). Best-corrected visual acuity was determined using the Snellen visual acuity chart. The primary outcome was the development of NVG in the ischemic or nonischemic CRVO eyes despite treatment. The secondary outcome was the development of ocular NV without NVG.

For statistical analysis, the Snellen visual acuity measurements were converted to logarithm of the minimum angle of resolution (logMAR) units before analysis [16]. Cirrus OCT measurements were approximated to Stratus OCT measurements by subtracting 60 microns from the central subfield measurement [17, 18]. SAS Version 9.2 was used for statistical analysis, and Fisher's exact test, chi-square test, independent* t*-test, and nonparametric Spearman's rank correlation were used. Statistical significance was set at *P* < 0.05. Statistical comparison of our results was performed against a historical control studying the probability of ocular NV and NVG after ischemic CRVO [19]; however, this did not yield statistically significant results due to our much smaller study group.

## 3. Results

This study included 44 eyes of 44 patients diagnosed with CRVO. Fourteen eyes of 14 patients had ischemic CRVO, and 30 eyes of 30 patients had nonischemic CRVO, with no bilateral cases (Table 1). Average age at presentation was 71 years in the ischemic group and 69.5 years in the nonischemic group. In our urban patient population, the ischemic group consisted of 31% black patients and 69% white patients. The nonischemic group consisted of 17% black patients, 67% white patients, and 3% Asian patients, while 13% declined to identify their race. Of the total 44 eyes, 42 eyes received bevacizumab; ranibizumab was given to 3 nonischemic eyes. During July 2012 through December 2012, aflibercept was delivered to 2 nonischemic eyes and 1 ischemic eye after these eyes had already been treated with bevacizumab. This trend toward bevacizumab rather than ranibizumab at our urban institution is almost certainly due to drug cost disparity and insurance coverage issues.

Patients with ischemic CRVO presented on average 14 days after symptom onset, compared with 24 days in the nonischemic group. Total mean follow-up duration was 23 months (range 8–41 months) for ischemic eyes and 24 months (range 8–64 months) for nonischemic eyes. Ischemic eyes received an average of 5.6 ± 5.1 doses (range 2–19 doses) of intravitreal anti-VEGF over a mean period of 12.1 months (range 4–31.6 months). Nonischemic eyes received an average of 8.4 ± 5.7 doses (range 1–23 doses) of anti-VEGF over a mean period of 18.0 months (range 2.6–47.5 months) (Table 2).

Nine of 14 eyes (64%) in the ischemic group had initial VA worse than 20/200, compared with 8 of 30 eyes (27%) in the nonischemic group (*P* = 0.021). Over the course of treatment, vision improved in 70% of nonischemic patients and 50% of ischemic patients (*P* = 0.197). Ocular neovascularization developed in 1 of 30 (3.3%) nonischemic eyes and 5 of 14 (35.7%) ischemic eyes (*P* = 0.002). Mean VA in the ischemic group was 20/400 on initial visit and 20/800 on final visit. Mean VA in the nonischemic group was 20/80 on initial visit and 20/50 on final visit. Mean initial central foveal thickness (CFT) on OCT in the ischemic group was 602 micrometers, with mean final CFT of 386 micrometers. The nonischemic eyes had a mean initial CFT of 488 micrometers, with mean final CFT of 293 micrometers.

Five of the 14 (36%) ischemic eyes developed ocular NV, at 16 months after symptom onset, on average. Specifically 2 eyes (14%) developed NVI at 13.2 and 10.5 months after symptom onset, 1 eye (7%) developed NVD at 17.8 months, and 2 eyes developed vitreous hemorrhage (VH) at 8.4 and 30 months, respectively (Table 3). Four of these 5 NV eyes had presenting vision worse than 20/200. Only four of the 5 eyes received PRP, delivered on average 12.8 months (range 8.4–17.8 months) after symptom onset and on average 12.1 months (range 8.2–17.3 months) after first anti-VEGF injection. The eye that did not receive PRP had a VH limiting the view for laser therapy. One of the NVI eyes progressed to NVG despite PRP, and both VH eyes progressed to NVG although one of the VH eyes had received PRP. Thus, 3 of the 14 ischemic eyes (21%) developed NVG, at a mean of 19.7 months after symptom onset.

The 5 ischemic eyes that developed ocular NV received a mean of 3.6 ± 1.1 injections (median 4, range 2–5 injections), whereas the 9 ischemic eyes that did not develop ocular NV received a mean of 6.8 ± 6.2 injections (median 4, range 2–19 injections) (*P* = 0.16). Only 1 (3%) of 30 nonischemic eyes developed ocular NV, specifically NVD. This eye received PRP and did not progress to NVG.

## 4. Discussion

Ischemic CRVO is associated with a high rate of ocular NV [4–6, 19] and, if left untreated, has a rate of NVG ranging from 23% to 60% [5, 19]. The greatest risk of anterior segment neovascularization occurs in the first six months after diagnosis of ischemic CRVO [6, 19, 20]. The development of ocular NV is probably most dependent upon the severity of retinal hypoxia [6]. The NVG associated with ischemic CRVO has historically been labeled “90-day glaucoma,” emphasizing the relatively early development of ocular NV and NVG after ischemic CRVO.

The 1997 Central Vein Occlusion Study (CVOS) [2], which enrolled eligible patients with vein occlusion of less than 1 year's duration, showed a high rate of early NVI development in ischemic CRVO eyes. The CVOS inception cohort consisted of 187 patients (26% of the whole group) examined within one month of symptom onset. In this cohort of patients that presented early in the course of the disease, 11 of the 26 ischemic eyes (42%) developed NVI or angle NV. Of the 187 cohort eyes, 20 of the 36 eyes (56%) with vision 20/200 or worse developed NVI or angle NV; thus, low vision at baseline in addition to perfusion status led to increased risk of NVI or angle NV. The median time to detection of NVI or angle NV was 61 days in the inception cohort. Among the larger group of all the included patients, the CVOS found that 35% of the 117 ischemic eyes developed angle or iris NV and received photocoagulation; however, 10 eyes (8.5%) progressed NVG. Since the CVOS, the standard of care for ischemic CRVO eyes is observation, with prompt PRP treatment if ocular NV develops.

The Clinical Trial of Subjects with Macular Edema Secondary to Central Retinal Vein Occlusion (CRUISE) [14] found that the anti-VEGF agent ranibizumab significantly improved macular edema and vision compared with observation only. The CRUISE sham group of 129 eyes had 11 eyes (8.5%) develop NVI during the first year of the study, and 2 eyes (1.6%) develop NVG within 6 months. In the treatment groups with ranibizumab 0.3 mg or 0.5 mg, 7 of 261 eyes (2.7%) developed NVI, and 1 eye (0.4%) developed NVG during the one-year time period. These patients received an average of 9.2 treatments in the one-year time period. However, the CRUISE study excluded eyes with a brisk RAPD, therefore likely excluding patients with severe ischemic CRVO [3, 14].

The one-year results from the Vascular Endothelial Growth Factor Trap-Eye for Macular Edema Secondary to Central Retinal Vein Occlusion (COPERNICUS) study [12] showed that aflibercept significantly improved macular edema and vision compared with observation. The patients, with CRVO diagnosis within 9 months of study screening, received monthly aflibercept or sham injection for the initial 6 months of the study then aflibercept as needed. Eyes with brisk RAPD were not excluded, and, in the sham group of 73 eyes, 23 (31.5%) eyes were ischemic or indeterminate. Two of these sham injection eyes (2.7%) developed NVI and another 2 eyes (2.7%) developed NVG within the first 6 months; an additional 1 eye developed NVG within the first year. In the aflibercept group of 114 eyes, which received a mean of 8.7 injections over 12 months, 27 (23,7%) eyes were ischemic or indeterminate; no eye developed anterior segment NV or NVG. Thus, the COPERNICUS study demonstrated that aflibercept reduced the rate of anterior segment NV and NVG. However, the COPERNICUS study results followed these eyes for only 12 months.

Another 2012 prospective randomized study [21] of 60 CRVO eyes investigated sham or bevacizumab injection every 6 weeks for the initial six months of the study. The study included patients with significant vision loss as low as 20/500, likely allowing for the inclusion of ischemic CRVO eyes. Five of the 30 sham eyes (17%) developed NVI by week 24, while none of the bevacizumab group developed NVI. After week 24, all 60 eyes in the study were treated with bevacizumab every 6 weeks. The NVI regressed in the 5 patients, and no patient in the study developed NVG. These findings suggest a protective effect of intravitreal anti-VEGF against ocular NV and NVG. The authors suggested that anti-VEGF treatments may simply delay the onset of neovascularization [21]. The study did not differentiate ischemic from nonischemic eyes, and follow-up was only for 12 months.

In our evaluation of the effect of anti-VEGF therapy on ischemic CRVO eyes, we compare our patients to Hayreh's natural history study [19] because we attempted to use similar criteria to differentiate between ischemic and nonischemic CRVO eyes. The CRUISE trial did not distinguish between ischemic and nonischemic CRVO and probably excluded the most severely ischemic CRVO eyes, which are the eyes of interest in our study. We included only patients who presented within 90 days of CRVO symptom onset, similar to Hayreh's study in which approximately 75% of eyes presented for initial evaluation within 90 days of CRVO onset [19]. The Hayreh study also had a similar follow-up duration, with 102 of 189 study eyes retained at 12 months and 75 of 189 eyes retained at 24 months.

Most of the eyes in our study received a sporadic combination of macular edema treatment between 2007 and 2012, including anti-VEGF, steroid injection, and focal laser, as determined by the treating retina specialist. Hayreh's natural history study [19] showed that the greatest risk of NVI occurs during the first 6 months after diagnosis, with a cumulative probability of NVI development of 49%, but not all eyes with iris or angle NV progress to NVG [19]. Our study results show that, compared to Hayreh's findings, ischemic CRVO eyes treated with anti-VEGF developed NVI at a reduced incidence (14%) and later time frame (11.9 months on average). However, this result should be interpreted with caution due to disparity in group size between our small study group of 14 eyes and the large natural history study group of 239 ischemic eyes [19].

Hayreh and Zimmerman [19] also found that ischemic CRVO was associated with a cumulative probability of 10% developing NVD, with the highest risk of NVD occurring within 12 months of onset. Our study had 3 of 14 eyes (21.4%) develop posterior segment NV in the form of vitreous hemorrhage (VH) or NVD, at 17.8, 30.0, and 11.9 months after symptom onset, despite anti-VEGF treatment. It is unclear why more eyes in our study developed posterior segment NV than anterior segment NV. Interestingly, Hayreh's natural history study found that NVD is most likely to develop in the first 12 months from onset, whereas, as stated above, NVI is most likely to develop within the first 6 months [19].

Three (21%) of the 14 ischemic CRVO eyes in our study developed NVG at a mean of 19.7 months, much later than that described in the natural history study by the CVOS [2] and Hayreh and Zimmerman [19]. Hayreh found the cumulative probability for development of NVG after ischemic CRVO to be 34% within 9 months of onset, with 89% of the NVG eyes developing NVG within 12 months of onset. Two of our three study eyes with NVG had received PRP at the time of ocular NV diagnosis yet progressed despite PRP. We suspect that the anti-VEGF treatment to these ischemic eyes may be the reason for the delayed development of NVG, but anti-VEGF and even subsequent PRP could not prevent NVG development because the severe ischemia persisted.

In our study, we compared the 5 ischemic eyes with ocular NV to the 9 ischemic eyes without ocular NV to determine if there were any identifiable factors leading the 5 eyes to develop NV. We found that there was no difference in the anti-VEGF frequency or the time period from symptom onset to anti-VEGF treatment initiation between these groups. The 9 eyes that did not develop ocular NV received on average 6.8 ± 6.2 total doses of anti-VEGF and the 5 eyes that developed ocular NV received 3.6 ± 1.1 total doses of anti-VEGF; the wide variation in the number of doses and the small group sizes precluded statistical analysis, yet there may be a trend toward fewer anti-VEGF doses administered to the 5 NV eyes. Probably the most ischemic eyes had less vision improvement with anti-VEGF doses for macular edema, and this led the treating retina specialist to abandon anti-VEGF for other treatment modalities or observation. The 30 nonischemic eyes in our study received significantly more anti-VEGF doses over a 24-month period than the 14 ischemic eyes (mean of 8.4 doses versus 5.6 doses, resp., *P* = 0.041). This is appropriate since the anti-VEGF was originally administered to treat macular edema, not in order to prevent the development of ocular NV and NVG.

The small sample size of 14 ischemic eyes is one of many limitations to this study. The four retina specialists who evaluated and managed the patients determined visit frequency and therapeutic intervention at their own discretion, resulting in varying treatment regimens. Gonioscopy was not routinely used to detect NV of the angle. It is possible that patients with angle NV but without iris NV were not identified in this study. However, previous studies showed that angle NV without iris NV [20] occurs at a significantly lower rate than iris NV [22]. Patients who received focal grid or intravitreal triamcinolone were not excluded from this study, with 8 of the 30 nonischemic patients and 5 of the 14 ischemic patients receiving triamcinolone, confounding the effect of anti-VEGF therapy on final visual acuity and retinal thickness on OCT. Although the SCORE study found that triamcinolone did not affect the rates of NVI and NVG among the different treatment groups [23], the effect of triamcinolone on intraocular VEGF remains to be determined [23–25].

The CRUISE and COPERNICUS trials demonstrate the effectiveness of anti-VEGF for macular edema resulting from CRVO, and the results from these studies imply that ocular NV and NVG are reduced from the administration of anti-VEGF, which is both logical and promising. Our study also suggests that anti-VEGF therapy can reduce ocular NV and NVG. However, as discussed above and as seen in the results of our study, anti-VEGF does not prevent ocular NV and may simply delay the development of ocular NV and NVG. Thus, significant risk of neovascularization remains, perhaps more so if anti-VEGF is delivered sporadically within the first one year of ischemic CRVO onset rather than on a monthly basis in the initial 6 months. Careful long-term monitoring for ocular NV is essential for these patients even while they are receiving anti-VEGF therapy. However, the advent of anti-VEGF agents for macular edema associated with both nonischemic and ischemic CRVO may have ended the era of “90-day glaucoma” secondary to ischemic CRVO.

## Figures and Tables

**Table 1:** Demographic and clinical features of patients with ischemic and nonischemic CRVO receiving anti-VEGF for macular edema.

Demographic or clinical variable	Nonischemic (*n* = 30)	Ischemic (*n* = 14)	*P* value
Age	69.5 (11.7)	71.0 (10.4)	0.722

Race			
Black	5 (16.7%)	4 (30.8%)	0.627
White	20 (66.7%)	9 (69.2%)	
Asian-Indian	1 (3.3%)	0	

Initial VA ≤20/200			
No	22 (73.3%)	5 (35.7%)	**0.021**
Yes	8 (26.7%)	9 (64.3%)	

VA change			
Improved/stable	21 (70.0%)	7 (50.0%)	0.197
Worsened	9 (30.0%)	7 (50.0%)	

Ocular NV (NVI, NVD, or VH)			
No	29 (96.7%)	9 (64.3%)	**0.002**
Yes	1 (3.3%)	5 (35.7%)	

**Table 2:** Ocular characteristics of ischemic and nonischemic CRVO eyes receiving anti-VEGF for macular edema.

Clinical variables	Ischemic (*n* = 14)	Nonischemic (*n* = 30)	*P* value
Total follow-up (mos), mean (SD)	23 (7.8)	24 (12.0)	
Time between symptom onset and first dose of anti-VEGF (days), mean (SD)	25 (19.6)	43 (50.0)	0.182
Total doses of anti-VEGF, mean (SD)	5.6 (5.1)	8.4 (5.7)	**0.041**
Interval between doses (mos), mean (SD)	2.2 (1.8)	2.0 (0.9)	0.558
Treatment duration (mos), mean (SD)	12.1 (8.0)	18 (11.3)	
Eyes with ocular NV, *n* (%)	5 (35.7) See Table 3	1 (3.3) NVD	
Duration from symptom onset to diagnosis of NV (mos), mean (SD)	16.7 (7.9)	9.3	
Eyes receiving PRP, *n* (%)	4 (28.6)	1 (3.3)	
Duration from symptom onset to PRP delivery (mos), mean (SD)	12.8 (3.9)	9.3	
Eyes with NVG, *n* (%)	3 (21.4)	0	
Duration from symptom onset to diagnosis of NVG (mos), mean (SD)	19.7 (9.9)	—	
Mean initial VA	20/400	20/80	
Mean final VA	20/800	20/50	
Mean initial CRT (mm), mean (SD)	602 (225)	488 (221)	
Mean final CRT (mm), mean (SD)	386 (254)	293 (145)	

**Table 3:** Ischemic CRVO eyes with ocular NV.

Patient	NV type	Total anti-VEGF doses received	Average interval between anti-VEGF doses (mos)	Duration symptom onset to NV diagnosis (mos)	Duration from symptom onset to PRP delivery (mos)	NVG	Duration from symptom onset to NVG diagnosis (mos)	Glaucoma surgery required
1	NVI	5	1.5	13.2	13.2	+	16.4	
2	NVI	4	1	10.5	11.7			
3	NVD	3	3	17.8	17.8			
4	VH	2	1.25	8.4	8.4	+	11.9	+
5	VH	4	5	30.0	Unable	+	30.8	
